# Supplementary figures and images for: Human, Nonhuman Primate, and Bat Cells Are Broadly Susceptible to Tibrovirus Particle Cell Entry
Source: Front Microbiol. 2019 Apr 26;10:856. doi: 10.3389/fmicb.2019.00856 (PMC6499107; doi:10.3389/fmicb.2019.00856)

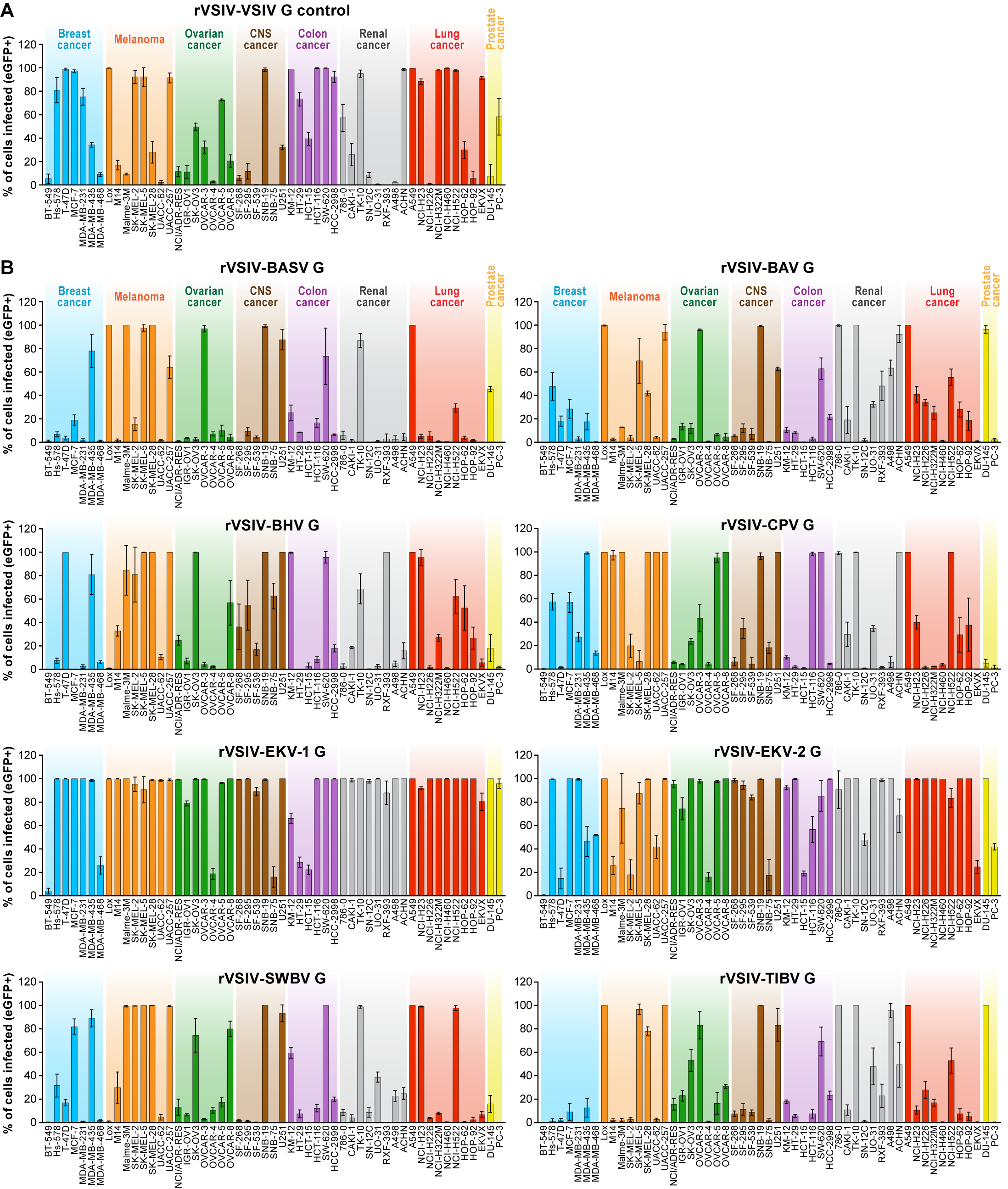

Supplement: FIGURE S1 — Tibrovirus glycoproteins mediate virion entry into a broad range of human cell types. (A) Same experiment as in Figure 1B and (B) as in 2 using rVSIVs expressing diverse tibrovirus glycoproteins (G) (MOI = 0.3). The percentage of eGFP-expressing NCI-60 human cell panel cell lines was measured by high-content imaging at 24 h post-exposure. All experiments were performed in triplicate; error bars show standard deviations. BHV, Beatrice Hill virus; BASV, Bas-Congo virus; BAV, Bivens Arm virus; CNS, central nervous system; CPV; Coastal Plains virus; EKV-1, Ekpoma virus 1; eGFP, enhanced green fluorescent protein; EKV-2, Ekpoma virus 2; MOI, multiplicity of infection; SWBV, Sweetwater Branch virus; TIBV, Tibrogargan virus; rVSIV, recombinant vesicular stomatitis Indiana virus. NCI-60 cell lines are listed by their abbreviations and grouped by organ/cancer type. [file Image_1.TIF]

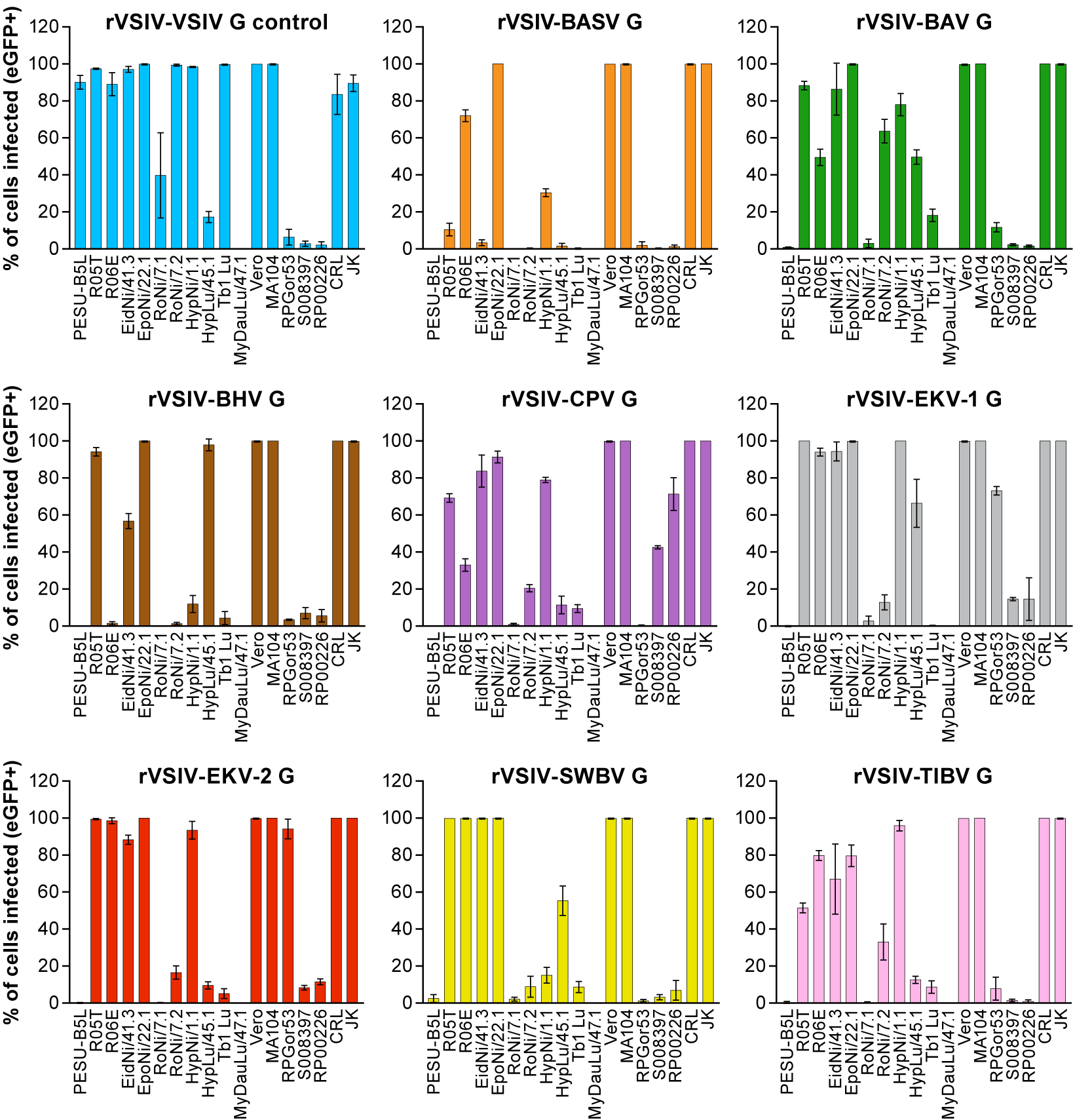

Supplement: FIGURE S2 — Tibrovirus glycoproteins mediate virion entry into a broad range of animal cell types. Same experiment as in Figure 3 using different cell types exposed to rVSIV–VSIV G control and rVSIVs expressing diverse tibrovirus glycoproteins (G) (MOI = 0.3). Bat (PESU-B5L, Ro5T, Ro6E, EidNi/41.3, EpoNi/22.1, RoNi/7.1, RoNi/7.2, HypNi/1.1, HypLu/45.1, Tb1 Lu, MyDauLu/47.1), nonhuman primate (Vero, MA104, RPGor53, S008397, RP00226), hispid cotton rat CRL, and boa constrictor JK cell lines. The percentage of eGFP-expressing cell lines was measured by high-content imaging at 24 h post-exposure. All experiments were performed in triplicate; error bars show standard deviations. BHV, Beatrice Hill virus; BASV, Bas-Congo virus; BAV, Bivens Arm virus; CPV, Coastal Plains virus; eGFP, enhanced green fluorescent protein; EKV-1, Ekpoma virus 1; EKV-2, Ekpoma virus 2; SWBV, Sweetwater Branch virus; TIBV, Tibrogargan virus; rVSIV, recombinant vesicular stomatitis Indiana virus. [file Image_2.TIF]
